# A systematic review and meta-analysis of the effects of non-pharmacological interventions on quality of life in adults with multiple sclerosis

**DOI:** 10.1186/s40001-023-01185-5

**Published:** 2023-08-22

**Authors:** Victor Gitman, Kasey Moss, David Hodgson

**Affiliations:** 1 London School of Hygiene and Tropical Medicine, London, UK; 2 Department of Medicine, McMaster University, Hamilton, Canada; 3 Centre for Mathematical Modelling of Infectious Diseases, London School of Hygiene and Tropical Medicine, London, UK

**Keywords:** Multiple sclerosis, Quality of life, Non-pharmacological therapies, Physical activity, Behavioral interventions, Psychological interventions, Systematic review, Meta-analysis

## Abstract

**Background:**

Multiple Sclerosis (MS) is a chronic debilitating disease that targets the central nervous system. Globally it is estimated that 2.8 million people live with MS (2018) and as there is no known cure; therefore, identifying methods to increase a patient’s quality of life (QoL) is of considerable importance. Non-pharmacological interventions are a viable and effective option to increase QoL in patients with MS, however, to date, the literature lacks a complete systematic review of these interventions.

**Methods:**

A literature search was conducted for studies published up until March 4th 2022 in Scopus, Web of Science, CINAHL Plus, The Cochrane Library, Medline, and Embase. Studies were included if they were randomized control trials (RCTs) assessing a non-pharmacological intervention in adults with MS and measured QoL using the MSQOL-54, SF-36 or MSQLI tools for at least two time points. Quality assessment of each study was completed as well as a review of publication bias. Where possible, meta-analysis was conducted using a random effects model and for other studies a qualitative synthesis was presented.

**Results:**

Thirty studies were included in the meta-analysis and eleven studies were summarized qualitatively. The pooled effects across all non-pharmacological interventions showed a modest improvement in both the physical and mental components of QoL, with a standardized mean difference (SMD) of 0.44 (95% CI 0.26–0.61) and 0.42 (95% CI 0.24–0.60), respectively. Non-pharmacological interventions based around a physical activity were found to be particularly effective in improving both the physical composite score (PCS) and mental composite score (MCS), with an SMD of 0.40 (95% CI 0.14–0.66) and 0.31 (95% CI 0.08–0.55), respectively. Interventions incorporating balance exercises presented a significant advantageous solution for improving QoL, with an SMD of 1.71 (95% CI 1.22, 2.20) and 1.63(95% CI 1.15–2.12) for PCS and MCS respectively.

**Conclusions:**

This systematic review and meta-analysis identified that non-pharmacological interventions can be an effective method of improving QoL in patients with MS, especially modalities with a physical activity component and balance interventions.

**Supplementary Information:**

The online version contains supplementary material available at 10.1186/s40001-023-01185-5.

## Background

Multiple Sclerosis (MS) is a chronic and debilitating neurological disease characterized by an individual’s immune cells attacking conductive myelin sheaths in the central nervous system (CNS) [1]. This leads to impairment in electrical nerve signaling, causing varying degrees of disability and neurodegeneration [1]. The risk of development and progression of MS can be decreased by modifying certain lifestyle factors including ultraviolet light exposure, vitamin D intake, weight loss, fish oil consumption and smoking cessation [2]. Globally, the mean age of diagnosis is 32 years old and there is no cure, meaning it is a lifelong condition affecting an individual’s peak productive years [3]. For this reason, treatment for MS is focused on ensuring that patients live a life of relatively good quality and maintain health in aspects important to them [4–6].

Health-related quality of life (HRQoL) is a concept used to represent a person’s perception of their health status [7]. It is a broad and holistic term that considers the physical, mental, social, and functional aspects of an individual's health at a point in time [7]. HR-QoL is measured through standardized tools and instruments, offering a quantitative method to monitor an individual’s health status in response to intervention changes over time. The MSQOL-54, SF-36 or MSQLI are widely validated and utilized tools for measuring HRQoL in MS [8–10]. MS patients find physical functioning, role limitation, vitality, general health, and the presence of bodily pain, predominant contributors to their QoL [10]. Furthermore, a patient’s perception of their own QoL is predictive of future disease progression and disability [3, 11].

Global MS prevalence has increased from 2.3 million in 2013 to 2.8 million in 2020, likely in part due to improved survival [12]. As MS prevalence increases, it is of critical importance to identify interventions that can increase the QoL of affected individuals [3]. This study aims to synthesize the available evidence and determine, quantitatively, the effect that non-pharmacological interventions have on QoL; with the goal of informing clinical practice and improving QoL in adults living with MS.

## Methods

### Search strategy

This systematic review and meta-analysis utilized the Preferred Reporting Items for Systematic Reviews and Meta-Analyses (PRISMA) recommendations [13]. A systematic search reviewed all peer reviewed articles in English from inception of the database until March 4, 2022. Scopus, Web of Science, Cumulative Index of Nursing and Allied Health Literature (CINAHL Plus), The Cochrane Library, Medline and Embase were searched. Articles were selected based on broad keywords identified within the title and abstract of the publications. The following keywords and Boolean search criteria were implemented (‘multiple sclerosis’ OR ‘encephalomyelitis disseminate’ OR ‘demyelinating’) AND (‘Health Status Questionnaire’ OR ‘SF-36’ OR ‘Multiple Sclerosis Quality of Life-54’ OR ‘MSQOL-54’ OR ‘Multiple sclerosis quality of life inventory’ OR ‘MSQLI’). The full search strings are available in Additional file 1 for each database. The search was limited to three validated QoL measurement tools, the Multiple Sclerosis quality of life inventory (MSQLI), MSQOL-54 and SF-36. The search was not expanded to other validated tools as they were judged to differ widely in aspects such as their complexity, aspects of QoL measured, completion time and recall period. Moreover, some of the commonly used MS specific QoL tools measure aspects that are not covered in others making them not directly comparable instruments [9].

### Inclusion criteria

All articles were independently evaluated by two reviewers to assess eligibility and disagreements were discussed, as required, with a third reviewer to decide. We only included articles which performed studies on adult patients with MS and did not focus on those with additional comorbidities (Table 1). If patients within the study had other comorbidities the study was still included unless the particular co-morbidity was an inclusion criterion to participate. Studies evaluating the impact of acute clinical care, such as the evaluation of nursing practices, were not included. We define ‘non-pharmacological’ as any intervention used to improve quality of life without a pharmaceutical or surgical modality (dietary supplements, vitamins and nutraceuticals were excluded from the definition of a pharmaceutical agent). We categorized non-pharmacological interventions into five broad categories; physical activity, behavioral or psychological, tissue manipulation, nutraceuticals/supplement, diet, and other non-pharmacological interventions.

**Table 1:** Summary of inclusion and exclusion criteria

Inclusion	Exclusion
English language	Not in English or partial content in English
Randomized control study published in peer-reviewed journals in any region of the world	Case studies, cross-sectional designs, cohort studies, quasi-experimental designs or cross-over designs
Non-pharmacological interventions	Pharmacological, acute clinical care, or Surgical interventions
Adults (≥ 18 y.o)	Children (< 18 y.o)
Patients with a clinical diagnosis of multiple sclerosis—all types and severities	Patients with additional comorbidities as specified in the inclusion criteria of the study
Studies measuring of Quality of Life at baseline and at least one time point after, for both arms utilizing MSQOL-54, SF-36 or the MSQLI	Studies not providing sufficient statistical data on pre- and post-QoL test scores such as SD, SE or *P*-values
Full text manuscript/publication	Abstracts, reviews, conference presentations

For the meta-analysis, we only included studies with a comparator arm including no intervention, usual/standard care (such as the continuation of a previous intervention), placebo, or a minor non-active intervention (such as education materials). Studies with multiple arms not containing a control group were reviewed and synthesized separately from the meta-analysis portion.

This review only included randomized control studies. For both the intervention and control arm, studies had to measure QoL at baseline and at another time point. Data on pre- and post-QoL scores such as mean, standard deviation (SD), standard error (SE), confidence intervals, or *P*-values had to be provided. Cross-over designs were not included due to (i) the potential issue of a carry-over effect which could confound the result and (ii) the difficulty in accounting for these long-term effects [14].

### Data extraction

The following information was extracted from the studies: study author, year of publication, country-region of the study, whether the study was conducted in a single center or multicenter, sample size, participants’ MS type, inclusion criteria for the Kurtzke Expanded Disability Status Scale (EDSS), mean age and range of participants, percentage of female participants, a description of the intervention and control, frequency and duration of the intervention, QoL measurement tool used, a statement summarizing the main results related to QoL changes, and metrics associated with the results (pre- and post-scores, mean difference, SD, SE, etc.). If QoL was measured at multiple time points after baseline only the last reading was extracted. The mean difference between two timepoints was computed as the arithmetic difference, and a pooled SD was calculated using the initial and follow-up time points assuming normal distribution. The pooled SD was calculated by summing the variances of each time point and taking the square root.

There were two main outcomes of interest related to the measurement tool post intervention (SF-36 and MSQOL-54); the reported change in the physical composite score (PCS) and the reported change in the mental composite score (MCS). The PCS is a representation of role limitations due to physical health, including bodily pain, energy/fatigue, sexual function, social function, and health distress, and the MCS is a representation of role limitations due to mental health, including overall quality of life, emotional well-being, social function and vitality [15].

### Data synthesis

Studies included in the meta-analysis portion of the review were assessed using the standardized mean difference between the intervention and control arm as the chosen effect size. For those studies not reporting a PCS score, the physical functioning score was used as it contributes the most weight to the scoring of the PCS [15, 16]. Similarly for the MCS score, the mental health score or emotional well-being subscale was used in cases where it was not available [15, 16].

A random effect model was selected to accommodate the likely heterogeneity in the populations included in the studies. The meta-analysis was stratified by the type of non-pharmacological intervention and done for both the PCS and MCS separately utilizing RevMan 5.4.1 [17]. A pooled effect for each non-pharmacological subgroup was calculated with 95% confidence intervals and a corresponding overall effect across all studies was also performed. An *I*^2^ statistic was calculated for each non-pharmacological intervention subgroup as well as overall to assess heterogeneity. *I*^2^ statistics of 0–40%, 30–60%, 50–90% and 75–100% were considered as; might not be important, moderate heterogeneity, substantial heterogeneity and considerable heterogeneity, respectively [18]. A sensitivity analysis was incorporated by excluding a study from each subgroup and examining its effect on the *I*^2^ statistic and SMD. Publication bias was analyzed and discussed by visual inspection of funnel plots. For those studies for which the mean was not within the 95% CI of the overall effect as per the forest plot, a *t*-test was performed using STATA 15.1, with a null hypothesis that the SMD in that study was equal to the overall/pooled standardized mean difference observed across all studies [19].

### Quality assessment

Studies included were evaluated using the Joanna Briggs Institute (JBI) critical appraisal checklist for randomized control trials (RCTs), a tool used to measure the methodological quality and risk of bias of a study [20, 21]. The completed JBI checklist is available in Additional file 2. Studies were deemed of insufficient quality and excluded if greater than 6 questions on the checklist were answered ‘No’.

## Results

### Literature search results

**Fig. 1:**
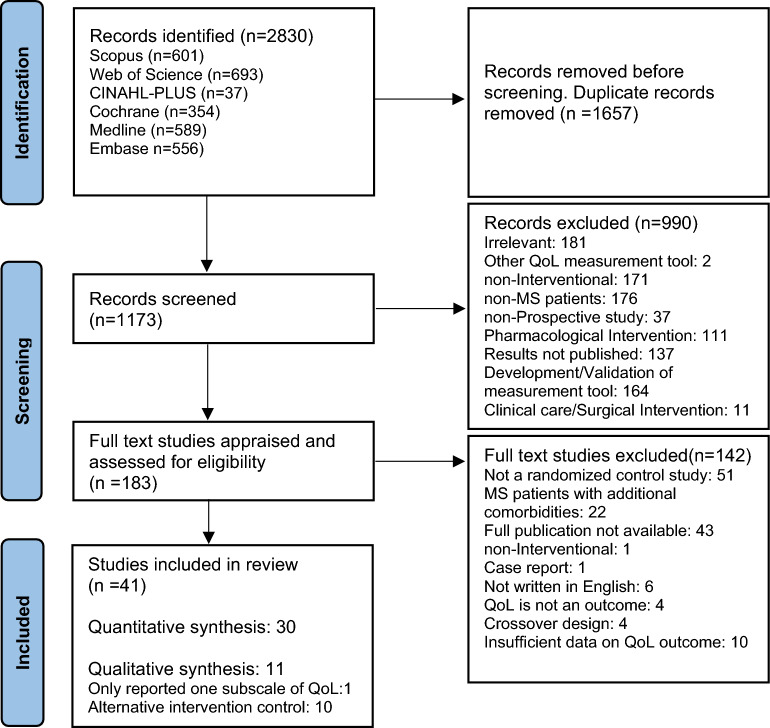
PRISMA flow diagram detailing the selection process for review and inclusion of studies into the systematic review and meta-analysis

A total of 2830 articles were identified from Scopus, Web of Science, CINAHL Plus, The Cochrane Library, Medline and Embase. Of these 1657 were identified as duplicate reports via Mendeley’s duplicate identification tool and through manual review. An additional 990 studies were excluded by two reviewers after reviewing the abstracts and titles. Full text review was conducted for the remaining 183 studies and of these 41 were deemed eligible to include in the current review (Fig. 1).

### Study characteristics

**Fig. 2:**
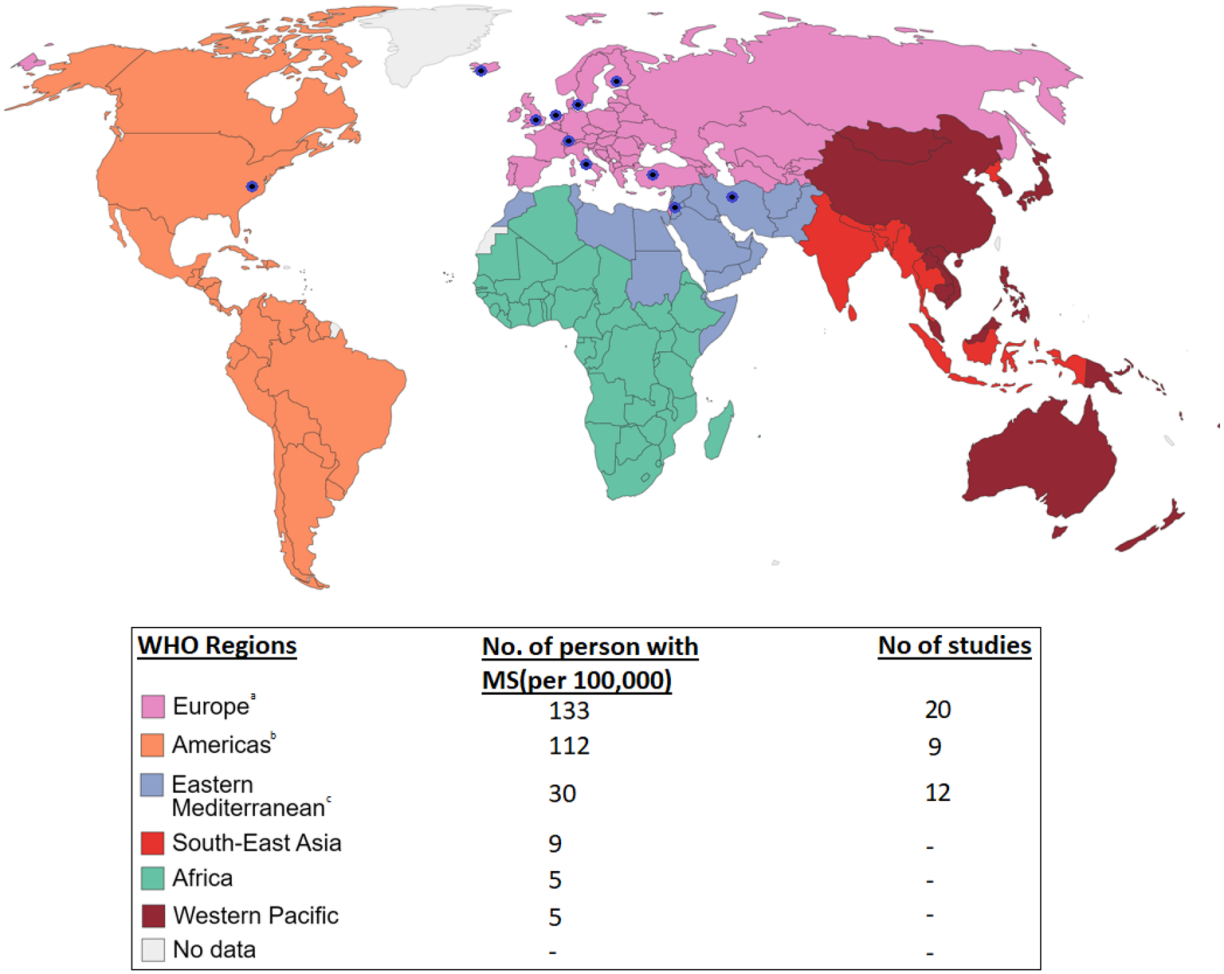
World map showing the six WHO regions [71] and the corresponding prevalence of MS [12] and number of studies included from each. ^a^There were 8 studies from Italy, 3 studies from the United Kingdom, 2 studies from Germany, 2 studies from Denmark, 1 study from Iceland, 1 study from The Netherlands, 1 study from Finland, 1 study from Switzerland, and 1 study from Turkey. ^b^There were 9 studies from the United States of America. ^c^There were 11 studies from Iran and 1 study from Jordan

For the quantitative review, there were 30 applicable studies identified published between 2002 and 2021. The duration of therapy ranged from 3 days to 6 months with 80% of studies being exposed to the non-pharmacological intervention for greater than 2 months. Sample sizes ranged from 11 to 169 participants and the total number of participants across all studies included was 2089. The most common study setting was Iran, 11, then there were 6 studies from America, 3 from the United Kingdom, 3 from Italy and the remaining 7 studies were done in Germany, Iceland, Netherlands, Denmark, Finland and Turkey (Fig. 2). 66% of studies used MSQOL-54, an MS-specific instrument to measure HRQoL and the rest utilized SF-36, a generic HRQoL measurement tool. The average age of participants was 42.1 years old and the average percentage of females in a study was 78% (range 54–100%). Most studies, 77%, either did not preclude participation based on their EDSS score or had inclusion criteria less than 5.5 indicating the patient’s ability to walk without aid or rest for 100 m [22]. The remaining 23% of studies had inclusion criteria with EDSS scores > 5.5 indicating the needs for assistance and a more severe disability [22]. Further details of each included study as well as a summary of their main results related to the QoL outcome are presented in Table 2.

**Table 2:** Summary of the characteristics of studies included in the meta-analysis investigating the effects of non-pharmacological interventions on health-related quality of life (HRQoL)—categorized by type of non-pharmacological intervention

Author/year	Region	Sample size (Ix/Cx)^a^	Participants	Mean age (range)	% Female	Intervention group	Control group	Duration	HRQoL tool	Summary of main outcome related to HRQoL
Physical activity interventions										
Ahadi 2013 [23]	Iran—Single Center	31 (10: Treadmill, 11: Yoga, 10: Control)	MS EDSS Score 1–4	34 (19–54)	100	Treatment 1: treadmill training consisted of 24 sessions of treadmill training (30 min), at 40–75% of age-predicted maximum heart rate. Treatment 2: Yoga group subjects participated in a thrice weekly 60–70-min sessions of Hatha yoga intervention	Followed own routine treatment program	8 weeks	MSQOL-54	The treadmill training program subjects showed a significant increase in the PCS. The yoga group also showed a significant increase in PCS (*P* = 0.02) and MCS (*P* = 0.00)
Ahmadi, Arastoo 2010 [24]	Iran—Single Center	20 (10, 10)	MS EDSS Score 1–4	37 (19–54)	100	Treadmill training (30 min), at 40–75% of age-predicted maximum heart rate—3 times per week	Wait-list control	8 weeks	MSQOL-54	In the control group, there was no significant change in any of the MSQOL-54 scale scores. Differences between the treadmill training group and the control group were statistically significant in 5 items of the MSQOL-54 scale scores (physical function, pain, energy, health perception and physical health composite)
Ahmadi 2010 [45]	Iran—Single Center	21 (11i, 10c)	MS EDSS Score 1–4	34 (19–54)	100	Hatha yoga classes 60–70 min 3 sessions per week	Wait-list control	8 weeks	MSQOL-54	Significant increase in some of MSQOL-54 scale scores in the yoga group (*P* ≤ 0.05). No changes for the control group. There was a significant difference found in both the PCS and MCS mean change between the intervention and control groups
Backus 2020 [25]	America—Atlanta—Single Center	12 (6, 6)	MS EDSS Score 7–8.5	55	58	Participants cycled volitionally with assistance from the electrical stimulation (Functional electrical stimulation: FES) as needed and with oversight for safety by the exercise staff. The goal was for participants to train three times a week	Wait list control	3 months	MSQOL-54	Significant increase in the physical health, health perception, health distress, and PCS in the training group
Barclay 2019 [26]	UK—Glasgow-Single Center	24 (15i, 9c)	MS EDSS Score 6–8.5	54	63	30 min of lower limb cycling programme using active passive trainers (2 min passive warm up, 26 min active cycling, 2 min passive cool down), 5 days per week	Usual care	5 weeks	MSQOL-54	Significant increase in both groups in both PCS and MCS. A larger effect size was demonstrated for the intervention group (0.93) and medium effect size in the control group (0.46)
Bjarnadottir 2007 [27]	Iceland	16 (6i, 10c)	MS EDSS Score < 4	37	63	Outpatient aerobic and strength exercise program (60 min) three times a week	Usual care—no change from previous	5 weeks	SF-36	Significant increase in vitality and a trend toward improved QoL in 5 of 8 parameters of SF-36
Carter 2013 [46]	UK—Sheffield—Single Center	30 (16i, 14c)	MS EDSS Score ≤ 5.5	40 (24–49)	87	Pragmatic exercise intervention (2 × supervised and 1 × home-based session per week)	Usual care	10 weeks	MSQOL-54	Significant increase in QoL which was sustained for up to 3 months after the intervention
Dalgas 2010 [33]	Denmark—Single Center	31 (16i, 15c)	MS EDSS Score 3–5.5	48	65	Progressive resistance training of the lower extremities performed twice weekly	Usual activity	3 months	SF-36	PCS QoL was increase significantly more in the intervention arm and this was maintained at follow-up after further 12 weeks
Hebert 2018 [35]	America—Colorado—Single Center	88 (44, 44)	MS	45	85	Balance and Eye-Movement Exercises for People with Multiple Sclerosis (BEEMS) was administered twice weekly with supervision and daily home exercise (phase 1) and in 1 supervised session weekly with daily home exercise (phase 2)	No treatment	4 months	SF-36	The BEEMS group showed a statistically greater increase in MCS compared to controls at 6 and 14 weeks
Jeong 2021 [32]	America-New York	45 (29i, 16c)	MS EDSS Score 5.5–7.5	57	73	Custom home exercise program with additional assistance by the telerehabilitation system	Usual care—custom daily home exercise plan	3 months	MSQOL-54	Patients in the telerehabilitation group showed significant improvement in pain and cognitive function symptoms in comparison with the control group
Kargarfard 2012 [47]	Iran—Isfahan	21 (10i, 11c)	RRMS (relapse remitting MS) EDSS Score ≤ 3.5	33	100	Supervised aquatic exercise in a swimming pool (3 times a week, each session lasting 60 min)	Usual care	8 weeks	MSQOL-54	Patients in the aquatic exercise group showed significant increases in QoL at 4 and 8 weeks compared with the control group
Langeskov-Christensen [34]	Denmark-multicentre	86 (43, 43)	MS EDSS score 0–6	45	60	Supervised progressive aerobic exercise (PAE) sessions with one continuous and one interval exercise session performed each week	Habitual lifestyle	24 weeks	SF-36	No differences in the SF-36 were found
Oken 2004 [36]	America—Oregon	57 (15: Exercise, 22: Yoga, 20: control)	MS EDSS Score ≤ 6	49	93	Group 1: weekly exercise class using a stationary bicycle along with home exercise Group 2: Weekly 90-min Iyengar yoga class along with home practice	Wait-list control	6 months	SF-36	Exercise and Yoga both significantly increase vitality on the SF-36 more as compared to the control group
Pappalardo 2016 [43]	Italy—Catania—Single Center	146 (49: outpatient, 49: inpatient, 48: control)	MS EDSS Score 4–8	46 (25–74)	64	Group A—Outpatient rehabilitation: once daily, 6 days per week, each session 60 min Group B—Inpatient rehabilitation: twice-daily, 6 day per week, each session 60 min	Wait-list control	5 weeks	SF-36	Outpatient rehabilitation significantly improved all sub scales of the SF-36 and was found to be more effective at improving QoL than inpatient rehabilitation
Patti 2002 [44]	Italy—Catania—Single Center	111 (58i, 53c)	MS EDSS Score 4–8	46 (25–60)	58	Outpatient rehabilitation program, 6 days a week	Wait-list control	6 weeks	SF-36	QoL increase significantly in the intervention group and this was seen at 6 and 12 weeks. The difference was significant between the intervention and control for all 8 subscales
Romberg 2005 [28]	Finland—Single Center	95 (47i, 48c)	MS EDSS Score 1–5.5	44	64	Progressive exercise program, mainly consisting of resistance training, it combined resistance training (3–4 times a week) with aerobic endurance training (once a week)	No treatment	6 months	MSQOL-54	“There was no effect seen in the MSQOL-54. The scores on the PCS and MCS of the MSQOL-54 were stable with no differences between groups”
Behavioral and psychological interventions										
De Giglio 2015 [37]	Italy—Rome—Single Center	35 (18i, 17c)	RRMS	44	74	Dr. Kawashima’s Brain Training (DKBT): How Old Is Your Brain video game 30 min/day, 5d/wk	No treatment	8 weeks	MSQOL-54	DBKT improved QoL significantly in the MCS, role limitations due to emotional problems, emotional wellbeing, and cognitive function when compared to the control
Jongen 2019 [48]	Netherlands—Single Center	158 (79, 79)	RRMS EDSS score ≤ 4	40	88	Intensive 3-day social cognitive treatment (can do treatment)	No treatment	3 days	MSQOL-54	In the intervention arm PCS and MCS were improved significantly at 1 month but this was not sustained at 3 and 6 months
Momenabadi 2019 [49]	Iran—Kermin—Single Center	80 (40, 40)	RRMS EDSS score ≤ 5	30 (20–35)	85	18 training sessions based on the main constructs of the health-promoting self-care behaviors system. Training class twice a week in 45–60 min sessions. In addition to holding in person training sessions, patients in the intervention group were followed up by phone calls and texts during the training period	No treatment	3 months	MSQOL-54	There was a significant increase in 14 subscales of QoL, MCS and PCS in the intervention arm and this was not observed in the control arm
O'Hara 2002 [38]	UK—Greater London	169 (73i, 96c)	MS	51 (28–81)	70	Self-care programme primarily comprised a discussion of self care strategies supported by an information booklet developed for the study in line with consumer priorities—2 discussion lasting 1–2 h over the month	No treatment	1 month	SF-36	There was a significant improvement in MCS in the intervention arm as compared to the control arm
Stuijbergen 2003 [39]	America—Community setting southwestern US	113 (56i, 57c)	MS	46 (25–60)	100	Lifestyle-change classes weekly for 90 min, then telephone follow-up	No treatment	8 weeks and then telephone follow-up for 3 months	SF-36	A significant improvement in the intervention arm was seen in mental health on the SF36 when compared to the control. There was no significant difference between the intervention and control for vitality, physical function, role–physical or role–emotional, social functioning, or general health
Nutraceutical/supplement interventions										
Ashtari 2016 [29]	Iran	94 (47, 47)	RRMS EDSS score ≤ 4	33	85	50,000 IU vitamin D3 every 5 days	Placebo	3 months	MSQOL-54	A significant increase in Mental Health QoL was seen in the intervention arm and this was not seen in the control arm
Etemadifar 2013 [50]	Iran—Isfahan—Single Center	54 (26, 26)	RRMS EDSS score < 5	34	100	250-mg Korean ginseng tablets twice daily after breakfast and evening meal	Placebo	3 months	MSQOL-54	A significant increase in most of the domains of the MSQOL were seen in the intervention arm as compared to the control arm
Namjooyan 2019 [40]	Iran—Khuzestan	51 (26i, 25c)	MS EDSS score 2–5.5	47	63	Two parts of ajwain and one part of Iranian borago were soaked in water for 24 h and one part of cinnamon was soaked in water for 72 h then were distilled—patients given 15 cc total per day (split in four capsules per day)	Placebo	3 months	MSQOL-54	A significant increase was seen in the physical and mental components of QoL in the intervention arm as compared to the control arm and this was sustained at 3 months
Nozari 2019 [30]	Iran—single centre	50 (25, 25)	RRMS	30	54	5 mg daily folic acid tablets and three divided doses of 1 mg injective vitamin B12	Placebo	2 months	MSQOL-54	A significant difference was seen in the physical and mental components of QoL in the intervention arm as compared to the control arm
Siahpoosh 2018 [51]	Iran—single centre	66 (33, 33)	MS	32	68	Capsules with powder of grape seed extract (plus excipients 50 mg) twice a day	Placebo	1 month	MSQOL-54	A significant difference was seen in the physical and mental components of QoL in the intervention arm as compared to the control arm
Diet interventions										
Moravejolahkami 2020 [41]	Iran—single centre	147 (68i, 79c)	RRMS EDSS score ≤ 3	39	83	A modified version of Mediterranean Diet (mMeD), based on higher intake of fresh fruits and vegetables, whole grains, monounsaturated fatty acids, fish, and low to moderate consumption of dairy products, meat, and poultry	Traditional Iranian diet	6 months	MSQOL-54	A significant increase was seen in the physical components of QoL in the intervention arm, however, this was not seen in the MCS
Tissue manipulation										
Doğan 2021 [52]	Turkey-multi center	66 (33, 33)	MS EDSS score < 5.5	38	NA	Reflexology was applied on each patient in the intervention group for 3 sessions a week	No treatment	3 months	MSQOL-54	A significant increase in the combined physical and mental health scores were found in the intervention group
Other interventions										
Shinto 2008 [31]	America—Oregon—Single Center	45 (15, 15, 15)	RRMS EDSS score ≤ 6	44	87	Intervention 1: naturopathic medicine which entailed 8 visits with a naturopath including daily supplements, Vitamins, diet intervention and counseling Intervention 2: MS education which entailed 8 visits with a nurse trained in MS care	Usual care	6 months	SF-36	“There were no significant differences between groups on any outcome measure”
Vermöhlen 2017 [42]	Germany—Multi Center	56 (25i, 31c)	MS EDSS 4–6.5	51	81	Hippotherapy once a week	Usual care	3 months	MSQOL-54	A significant increase was seen in the PCS and MCS in the intervention arm

^a^In the sample size column ‘i’ refers to the intervention arm and ‘c’ is in reference to the control arm

### Methodological quality and risk of bias

**Fig. 3:**
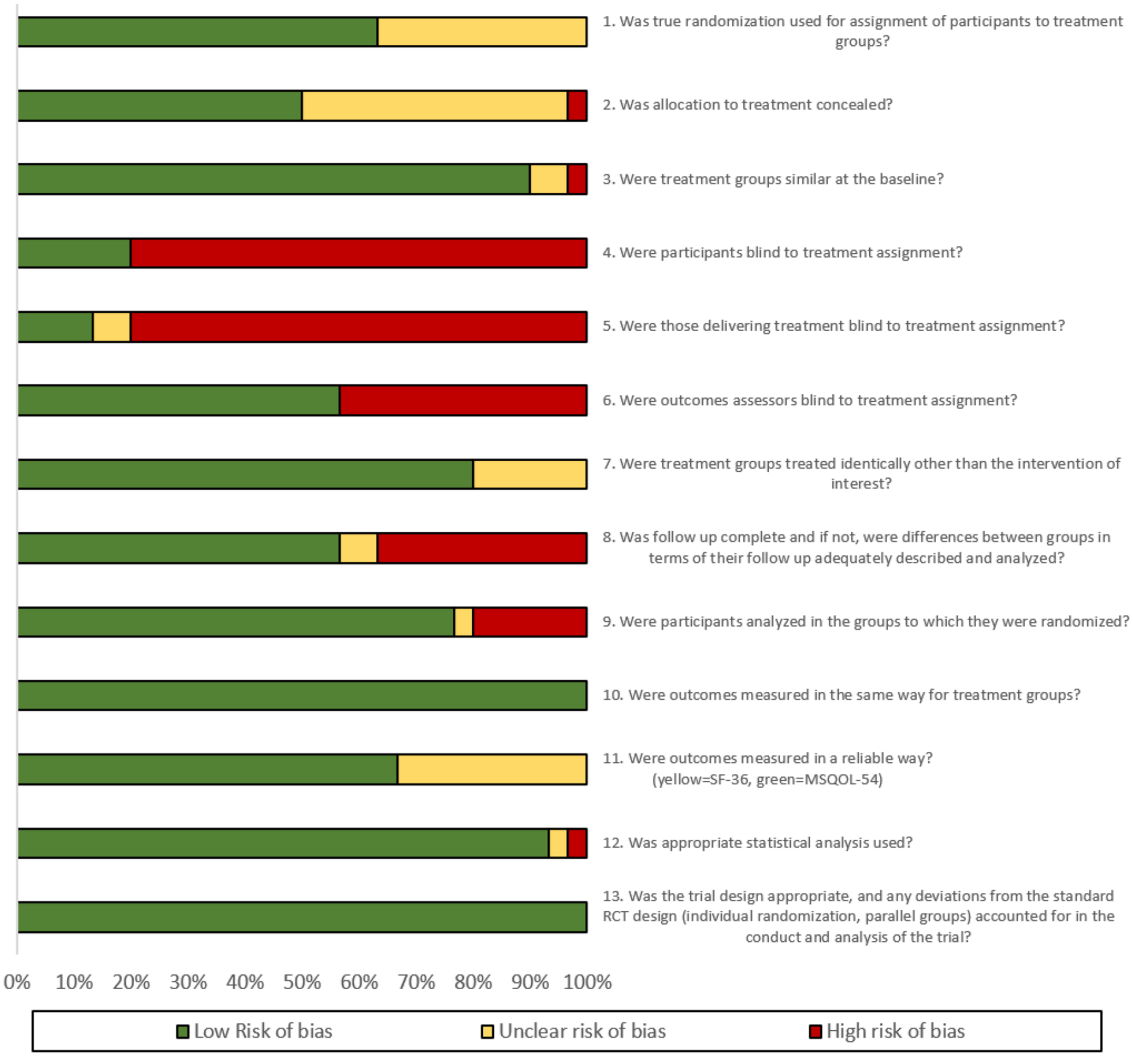
Summary of methodological quality and risk of bias based on the JBI critical appraisal checklist for RCTs across all studies included in the meta-analysis

A full review of the methodological quality and risk of bias for the 30 RCTs included in the meta-analysis is summarized in Fig. 3, further details are available in Additional file 2. Although all studies are RCTs, it was unclear if true random assignment was utilized for 11 of the studies [23–32]. The control arms and intervention arms were similar at baseline for 27 out of 30 studies. Differences between the intervention arm and control arm were in the majority (80%) of studies only attributable to the intervention assignment and not other factors such as baseline characteristics or follow-up frequency. For 11 studies there were differences between arms in the number of withdrawals and incomplete outcome data, and these differences were not sufficiently described [27, 33–42]. However, 23 studies did conduct an intention-to-treat analysis which examined the effects based on the initial allocation of a participant. Namjooyan et al. [40] did not calculate SD correctly and this was recalculated based on the confidence interval presented. No studies were excluded on the basis of low methodological quality or a high risk of bias.

### Effects of non-pharmacological intervention included in the meta-analysis

**Fig. 4:**
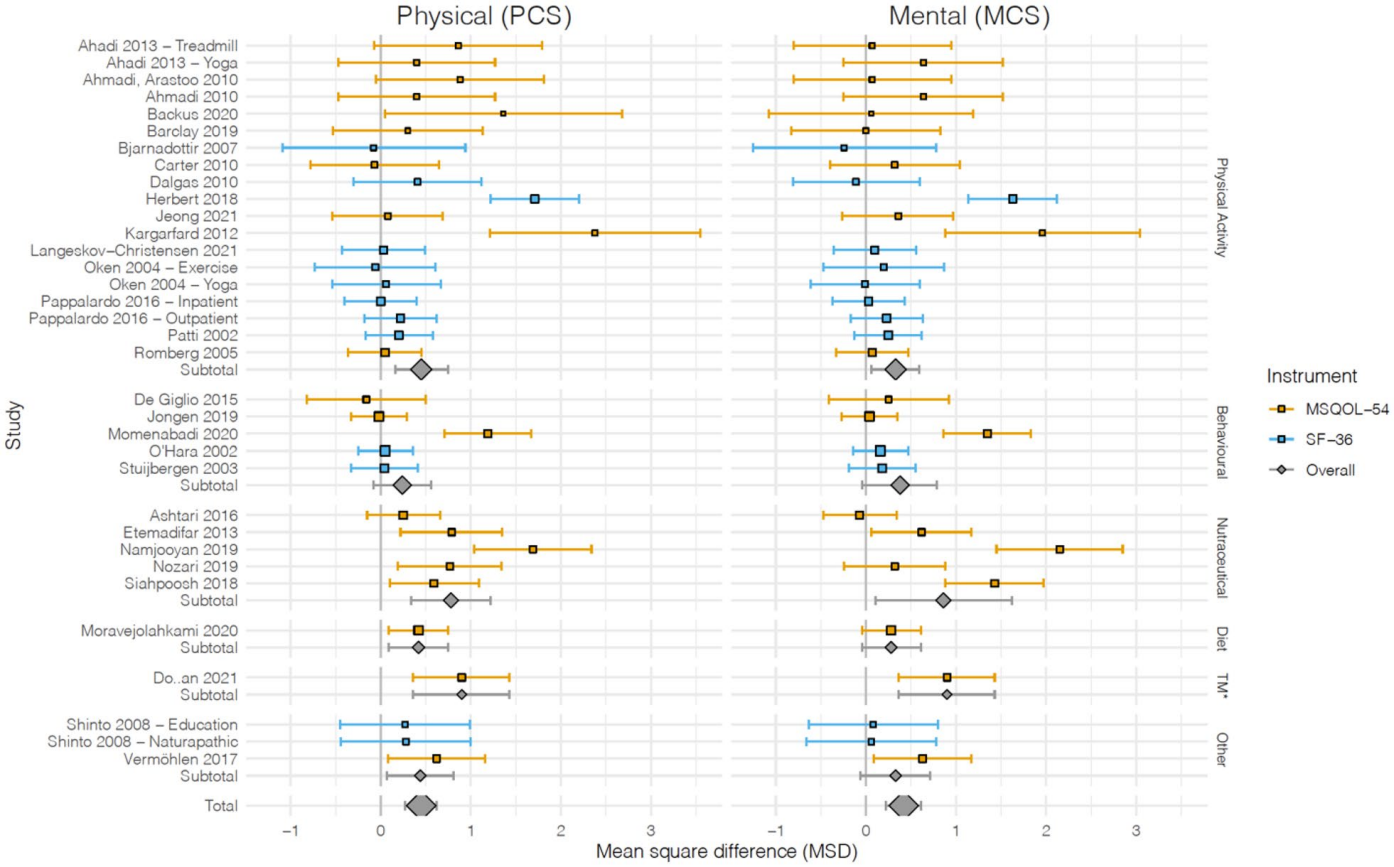
Forest plots of non-pharmacological intervention effect on the physical health (left column) and mental health (right column) component of health-related quality of life domains. Point estimates indicate the mean and error bars indicate the 95% confidence interval. Blue and orange indicate that the study utilized the SF-36 and MSQOL-54 measurement tool, respectively. The mean value pointer size is scaled according to the sample size of each study

There were 16 physical activity intervention studies in the meta-analysis, and activities involved rehabilitations programs [43, 44], yoga [23, 36, 45], aerobic and strength exercises [26–28, 32–34, 46], aquatic exercise [47], balance and eye-movement exercises [35], treadmill training and cycling programs [24, 25, 36] (Fig. 4). The physical component of QoL saw an overall positive effect with an SMD of 0.40 (95% CI 0.14, 0.66), however, substantial heterogeneity was observed (*I*^2^ = 69%, *P* < 0.001). Hebert et al. [35] which utilized a specifically created regiment of balance and eye-movement exercises for people with MS (BEEMS) for 4 months showed a larger SMD of 1.71 (95% CI 1.22, 2.20). Kargarfard et al. [47] which utilized a supervised aquatic exercise program for 8 weeks also showed a larger SMD of 2.38 (1.21, 3.55). Performing a sensitivity analysis by excluding Hebert et al. still showed a significant pooled effect of physical activity interventions on the PCS, SMD 0.24 (95% CI 0.06, 0.42) with an (*I*^2^ = 32%, *P* = 0.09). An overall positive effect was also seen on the mental component of QoL with an SMD of 0.31 (95% CI 0.08, 0.55), however, substantial heterogeneity was observed (*I*^2^ = 62%, *P* < 0.001). Hebert et al. [35] and Kargarfard et al. [47] showed a larger SMD of 1.63 (95% CI 1.15, 2.12) and 1.96 (95% CI 0.88, 3.04) respectively. A sensitivity analysis excluding Hebert et al. still showed a significant pooled effect of physical activity interventions on the MCS, SMD to 0.18 (95% CI 0.04, 0.32) with an (*I*^2^ = 0%, *P* = 0.52). The changes in the *I*^2^ statistics indicate that most of the heterogeneity was likely due to Hebert et al. for both PCS and MCS.

There were 5 behavioral and psychological interventions studies in the meta-analysis and activities included brain training programs [37], a short social cognitive treatment [48], self-care programs and lifestyle change classes [38, 39, 49]. The physical component of QoL did not see a significant overall effect with an SMD of 0.22 (95% CI − 0.19, 0.62) with substantial heterogeneity observed (*I*^2^ = 81%, *P* < 0.001). Momenabadi et al. [49] which investigated training sessions educating participants on health-promoting self-care behaviors lasting 4 months showed a significant effect on PCS, SMD of 1.19 (95% CI 0.71, 1.67). A sensitivity analysis excluding Momenabadi et al. still showed no significant pooled effect of behavioral/psychological interventions on the PCS, SMD 0.01 (95% CI − 0.17, 0.19) with an (*I*^2^ = 0%, *P* = 0.94). A significant overall effect was also not seen on the mental component of QoL with an SMD of 0.38 (95% CI − 0.04, 0.79) with substantial heterogeneity observed (*I*^2^ = 81%, *P* ≤ 0.001). Momenabadi et al. also showed a large significant effect on MCS, SMD of 1.35 (95% CI 0.86, 1.83). A sensitivity analysis excluding Momenabadi et al. still showed no significant pooled effect of behavioral/psychological interventions on the MCS, SMD 0.13 (95% CI − 0.05, 0.31) with an (*I*^2^ = 0%, *P* = 0.91). The changes in the *I*^2^ statistics indicate that almost all of the heterogeneity was likely due to Momenabadi et al. for both PCS and MCS.

There were 5 nutraceutical and supplements studies in the meta-analysis and included vitamin D [29], Korean ginseng tablets [50], folic acid tablets & vitamin B12 injections [30], grape seed extract capsules [51] and a traditional formulation containing cinnamon, ajwain and Iranian boragom [40]. The interventions ranged in duration from 1 to 3 months. An overall positive effect was seen on the physical component of QoL with an SMD of 0.78 (95% CI 0.34, 1.22), however, substantial heterogeneity was observed (*I*^2^ = 71%, *P* = 0.007). Ashtari et al. [29] was the only study within the subgroup that did not show a significant effect on PCS, SMD of 0.25 (95% CI − 0.15, 0.66). Performing a sensitivity analysis by excluding Namjooyan et al. [40] still showed a significant pooled effect of nutraceutical/supplement interventions on the PCS, SMD 0.54 (95% CI 0.28, 0.80) with an (*I*^2^ = 9%, *P* < 0.001). The change in the *I*^2^ statistic indicates that most of the heterogeneity was likely due to Namjooyan et al. An overall positive effect was also seen on the mental component of QoL with an SMD of 0.86 (95% CI 0.11, 1.62), however, considerable heterogeneity was observed (*I*^2^ = 90%, *P* < 0.001). A sensitivity analysis was done but no single study contributed significantly to the heterogeneity in MCS. Korean ginseng, the traditional formulation containing cinnamon, ajwain and Iranian boragom, and grape seed extract capsules all showed a significant effect on the mental health component of QoL; SMD 0.62 (95% CI 0.062, 1.17), SMD 2.15 (95% CI 1.45, 2.85), and SMD 1.45 (95% CI 0.88, 1.97), respectively.

For interventions involving a change in diet, only one study was included in the meta-analysis and involved a modified Mediterranean diet, lasting 6 months [41]. A positive effect was seen on the physical component of QoL with an SMD of 0.42 (95% CI 0.09, 0.75). A significant effect was not seen on the mental component of QoL with an SMD of 0.28 (95% CI − 0.04, 0.61).

There was one study included as part of the tissue manipulation category and involved reflexology, lasting 3 months [52]. A positive effect was seen on both the physical component of QoL with an SMD of 0.79 (95% CI 0.26, 1.32) and the mental component of QoL with an SMD of 0.90 (95% CI 0.36, 1.43).

Other interventions included a naturopathic medicine regimen [31], MS education [31], and hippotherapy [42]. A positive effect was seen on the physical component of QoL with an SMD of 0.44 (95% CI 0.07, 0.81). Only the Vermohlen et al. study [42], which utilized hippotherapy once a week for 3 months, showed a significant improvement in PCS, SMD of 0.62 (95% CI 0.08, 1.16). A significant overall effect was not seen on the mental component QoL with an SMD of 0.33 (95% CI − 0.06, 0.71). However, the Vermohlen et al. study showed an improvement in MCS, SMD of 0.63 (95% CI 0.09, 1.17).

### *t*-Tests comparing individual study effects and the overall pooled effect estimate

For the effect of non-pharmacological intervention on the physical health component of QoL there were 4 studies for which the SMD was outside the 95% CI of the pooled overall effect (Fig. 4) [35, 40, 47, 49]. For each of these studies a *t*-test was performed with a null hypothesis that the SMD was no different than the overall pooled SMD of 0.44 (SD: 4.08). Hebert et al. and Kargarfard et al. both showed strong evidence against the null hypothesis (*P*-value = 0.0042 and 0.0439 respectively). Namjooyan et al. and Momenabadi et al. showed weak evidence against the null hypothesis and it is possible that the difference in SMD was due to chance (*P*-value = 0.0692 and 0.4323, respectively).

For the effect of non-pharmacological intervention on the mental health component of QoL there were 5 studies for which the SMD was outside the 95% CI of the pooled overall effect (Fig. 4) [35, 40, 47, 49, 51]. For each of these studies, a *t*-test was performed with a null hypothesis that the SMD was no different than the overall pooled SMD of 0.42 (SD: 4.20). Hebert et al. showed strong evidence against the null hypothesis (*P*-value = 0.0085). Siahpoosh et al. (*P*-value = 0.0955), Namjooyan et al. (*P*-value = 0.0525), Momenabadi et al. (*P*-value = 0.3718) and Kargarfard et al. (*P*-value = 0.1455) showed weaker evidence against the null hypothesis and it is possible that the differences in SMD were due to chance.

*Summary of studies not included in meta-analysis—qualitative synthesis:* there were 11 studies not included in the quantitative synthesis which are described in detail in Additional file 3. Seven of these studies compared physical regiments [53–59], two studies compared behavioral/psychological regimens [60, 61], one study (Weinstock-Guttman et al.) compared supplements [62] and one study examined the effects of transcranial random noise stimulation (tRNS) (Salemi et al.) [63]. Nine studies showed an improvement in some aspects of QoL, with the exceptions of Pilutti et al. [58] and Plow et al. [61]. Of these, five studies did not show a statistically significant difference in the effect between the groups. Of note Khalil et al. [55] considered the impact of a virtual reality exercise program two times per week and 1 balance exercise session at home or traditional balance exercises at home without virtual reality over a period of 6 weeks. The results showed that the arm incorporating virtual reality had a significantly larger effect on both PCS and MCS than traditional balance exercises (*P*-value < 0.05)[62]. Solari et al. [59] considered the impact of an inpatient rehabilitation program consisting of daily exercises two times per day lasting 45 min or a home exercise program for 3 weeks. The inpatient rehabilitation group improved significantly more in the MCS and this was sustained at 3 and 9 weeks (*P*-value = 0.001) [66]. Impellizzeri et al. [60] considered the impact of a conventional cognitive rehabilitation (CCR) and neurologic music therapy (NMT) or just CCR 6 times per week for 8 weeks. Results showed a significantly greater improvement in mental health in the CCR and NMT group as compared to the only CCR group (*P*-value < 0.001) [67]. Salemi et al. [63] considered the impact of tRNS applied 15 min daily for 2 weeks or a sham control group [70]. The results showed a significant increase in the tRNS group in both the ‘change in health’ and role limitation due to physical problems’ subscales, *P*-value = 0.006 and 0.001, respectively [63].

### Publication bias

The funnel plot of the studies reporting a PCS outcome is presented in Additional file 4. There is asymmetry in the plot implying that there is the possibility of publication bias [71]. The gap in the bottom left corner of the plot indicates that studies with a larger SE which found no significant effect on PCS may have gone unpublished [64]. The funnel plot of the studies reporting an MCS outcome is also presented in Additional file 4. This plot is more symmetrical around the mean SMD compared to the PCS outcome funnel plot, implying that there may have been some studies which found a strong negative effect but went unpublished [64].

## Discussion

This is the first systematic review incorporating a meta-analysis to examine the effects of non-pharmacological measures on improving HRQoL in adults with MS. This study found that overall, there was a modest improvement in both the PCS and MCS of QoL measures across all studies incorporating non-pharmacological measures (Fig. 4). Important heterogeneity was observed, however, when stratified by type of non-pharmacological measure and performing sensitivity analysis the source of heterogeneity was elucidated. The results of this study build on previous work on this topic which qualitatively summarized the benefits of psychological and behavioral interventions for improving QoL [10].

### Principal findings

Across the 30 studies included in the meta-analysis there was a moderate effect for both the PCS and MCS on QoL for physical interventions. Although these results had a high risk of heterogeneity, most was contributed by a single study [Hebert et al.] and when this was removed a moderate effect was still observed for both PCS and MCS. Aquatic exercise [47] and balance and eye movement exercises (BEEMS) [35] showed a larger effect on PCS and MCS than the pooled effect within the physical intervention category (Fig. 4). When a *t*-test was performed comparing the SMD of individual studies and the overall pooled SMD in PCS, BEEMS and aquatic exercise both showed a statistically significant result indicating high probability that the difference was not due to chance. BEEMS also showed a significantly different effect on the MCS as compared to the overall pooled effect. BEEMS is a promising finding as previous studies have also shown a positive effect on patients with MS in aspects such as fatigue, posture control and disability [65–68]. Dizziness and instability are significant symptoms in patients with MS [69] and perhaps improving these over the course of the 4-month BEEMS program contributed greatly to the larger increase in MCS/PCS observed as compared to physical interventions which focused exclusively on either aerobic or resistance training.

Behavioral and psychological interventions did not show an overall pooled effect on PCS or MCS. Although there was a substantial amount of heterogeneity, it was mostly contributed by one study, Momenabadi et al. [49]. Conversely, the results of a review conducted by Gil-Gonzalez et al. [10] did comment on an effect of these types of interventions on QoL. This was a surprising finding, however, although some of the studies did report a significant effect on MCS in the intervention arm, not all studies included in the review by Gil-Gonzalez [10] included a control arm and this may have contributed to this apparent discrepancy.

Nutraceuticals and supplements overall showed an improvement in the PCS and MCS even after accounting for heterogeneity between studies, although the source of heterogeneity was unable to be determined for the effects on MCS. Therefore, it is likely that this subgroup is a good option to increase PCS and a possible one to improve MCS in patients with MS. Caution is recommended when generalizing these findings as all studies in this subgroup were done in a single country, Iran. The results in the diet intervention group, involving a modified Mediterranean diet, showed a moderate effect on PCS and no effect on MCS. This result needs to be interpreted with caution as the control arm was a traditional Iranian diet [41] which may differ substantially from other diets around the world such as the typical western diet. The tissue manipulation category involving reflexology demonstrated a benefit to both PCS and MCS. However, the tissue manipulation subgroup only included one single country study with a small sample size [52]. Thus, the conclusions of the study may not be widely generalizable and require further investigation.

The remaining studies not falling into any other categories were naturopathic medicine regimen [31], MS education [31], and hippotherapy [42]. The results were pooled, however, the interventions varied substantially and thus do not warrant discussion as a summary estimate. Hippotherapy showed a positive improvement in PCS, SMD of 0.62 (95% CI 0.08, 1.16) and although this is only one study a previous non-RCT also showed a positive effect of hippotherapy on aspects such as pain, muscle tension and balance [70]. Thus, it would be worthwhile for future studies to further explore the efficacy of hippotherapy on MS and QoL.

The results of the qualitative review, including 11 studies, were complimentary to that of the meta-analysis and quantitative synthesis. The Gandolfi et al. [54] study which incorporated SIBT in one of the arms provides further evidence for the effectiveness and importance of balance training for people with MS. Khalil et al. and Munari et al. both incorporated virtual reality (VR) into the physical intervention and had positive outcomes on QoL [55–57]. Pilutti et al. which investigated a stepper training program in one arm and a treadmill training in the other arm found no significant effects on any QoL outcome for either of these interventions [58]. It is possible, however, that no effect was observed due to the fact that all patients had progressive MS and were significantly disabled (EDSS score 6–8). This may indicate that perhaps exercise interventions may diminish in effectiveness in those with a more disabling disease status. Impellizzeri et al. added neurologic music therapy (NMT) to conventional cognitive rehabilitation (CCR) and this showed a greater improvement in mental health indicating the need to further explore NMT [60]. Another unique intervention was transcranial random noise stimulation (tRNS), a form of non-invasive brain stimulation, that showed statistically significant improvements in the ‘change in health’ and ‘role limitation due to physical problems’ subscales [63]. tRNS also needs to be further studied in MS patients with a larger sample size to more concretely understand its effects on QoL.

### Quality of the evidence

The systematic review was successful in identifying 1173 studies for screening which speaks to the wide and broad search criteria. This led to 41 RCTs of similar design to be included in this review which allowed for the ability to make robust and useful discoveries. There was minimal selection bias introduced by restricting the review to SF-36, MSQLI, MSQoL-54 as these are the most widely used and comparable measure of HR-QoL in MS. Non-pharmacological studies were of similar duration, with 77% being administered for 2 months or greater which allowed for a fair comparison to be made across interventions and provided further support to pool the results. Also, the studies included in the meta-analysis were overall representative of the global MS population, with studies covering 10 diverse countries (Fig. 2) [71] and being majority female in representation (range 54–100% female in studies compared to 69% globally). The mean age of a participant in this review at 42.1 years old with a SD of 7.7 was comparable to the global mean age of MS diagnosis of 32 years old [3]. There was likely some selection bias within the studies; this is because for 11 of the studies we are not certain if true randomization was used and for 15 studies it was unclear if allocation was concealed. For those studies administering a nutraceutical/supplement sufficient blinding of participants was maintained using a placebo [29, 40, 50, 51]. However, in 13 studies those assessing QoL were not blind to assignment, and this may have introduced detection bias overestimating the results [23, 25, 28, 33, 34, 39, 45, 48, 49, 51, 52, 72]. In addition, for many studies it was not feasible to blind participants to the interventions and this may have contributed significant bias and led to an overestimation of the true benefit. Confounding was not deemed to be an issue in this study as potential confounders such as age or EDSS score were controlled for via randomization. Finally, it is also possible based on visual inspection of the funnel plots that publication bias was present and thus studies that showed a negative effect of non-pharmacological interventions went unpublished. However, this may have been due to heterogeneity in the interventions, for example, the majority of nutraceutical/supplement studies showed a statistically significant effect on PCS whereas the majority of behavioral/psychological studies showed no statistically significant effect on PCS. Overall, the quality of the studies was good as all were RCTs with similar patient characteristics at baseline and the majority, 23, conducted an intention-to-treat analysis. No studies were excluded from this review on the basis of quality.

### Limitations

Studies were limited in size with a mean sample size of 67 patients, and this can affect the power of the studies and uncertainty of the effect size. Additionally, most studies were conducted at a single center making it difficult to generalize the outcomes to a wider region or country. There was also some heterogeneity in the disability status of patients across studies. This is noteworthy, as those with more disabling disease may be less likely to derive benefit from certain interventions and conversely those with less disabling disease may be less likely to derive benefit from interventions designed for those with a higher EDSS score. The duration of therapy and follow-up was overall quite short and given this is a chronic condition one cannot make claims that the benefits seen will be maintained over longer periods. Lastly, it should be noted that this review was not prospectively registered. 

## Conclusion

This systematic review and meta-analysis found that overall, non-pharmacological interventions improve QoL in persons with MS and that physical activity is particularly important. Specifically, balance exercises present a significant advantageous solution for improving both the mental and physical components of QoL. Other modalities such as nutraceuticals and supplements, diet and hippotherapy were found to improve either the PCS or MCS and behavioral and psychological interventions did not show an improvement in either the PCS or MCS. Comparing the pooled estimates of non-pharmacological intervention types needs to be done with caution as each subgroup did not contain an equal number of studies. Thus, we cannot draw definitive conclusions with regards to comparing the pooled effects. Further, this review looked only at changes in composite scores, this does not preclude that a significant change in overall QoL may have been seen or a change in one subscale may have been observed. Future studies on this topic should examine; all subscales of the QoL tools as well as the PCS and MCS, only include studies for which the inclusion criteria specified an EDSS score of < 5.5, and examine interventions over a longer duration of time. Also, additional studies need to be done to provide more evidence and clarify the true effects of nutraceuticals and supplements, diet, hippotherapy and behavioral and psychological interventions on QoL. Although there are still gaps in the existing literature to draw definite conclusions, this work demonstrates that non-pharmacological interventions improve QoL in persons with MS and that physical activity is likely to be very important. Moreover, many of the interventions discussed have few risks to implementation and thus represent a real and viable solution for improving QoL in persons with MS.

### Supplementary Information

**Additional file 1.** Detailed search strategy.

**Additional file 2.** Complete JBI critical appraisal checklist for all studies included in the meta-analysis.

**Additional file 3.** Qualitative synthesis of studies not included in the meta-analysis.

**Additional file 4.** Funnel plots of studies included in the meta-analysis.

## Data Availability

All data generated or analyzed during this study are included in this published article and its Additional files.

## Abbreviations

BEEMS: Balance and eye-movement exercises for people with MS
CCR: Conventional cognitive rehabilitation
CI: Confidence interval
CINAHL: Cumulative Index of Nursing and Allied Health Literature
CNS: Central nervous system
DKBT: Dr. Kawashima’s Brain Training
EDSS: Kurtzke Expanded Disability Status Scale
FES: Functional electrical stimulation
HRQoL: Health-related quality of life
JBI: Joanna Briggs Institute
LSHTM: London School of Hygiene and Tropical Medicine
MCS: Mental composite score
mMeD: Modified Version of Mediterranean Diet
MS: Multiple sclerosis
MSIS-29: Multiple sclerosis impact scale
MSQLI: Multiple sclerosis quality of life inventory
MSQOL-54: Multiple sclerosis quality of life-54
MusiQoL: Multiple Sclerosis International Quality of Life questionnaire
NMT: Neurologic music therapy
PCS: Physical composite score
PRISMA: Preferred Reporting Items for Systematic Reviews and Meta-Analyses
QoL: Quality of life
RAGT: Robot assisted gait training
RCT: Randomized control trial
RRMS: Relapse remitting multiple sclerosis
SD: Standard deviation
SE: Standard error
SF-36: Short form health survey
SIBT: Sensory integration balance training
SPMS: Secondary progressive multiple sclerosis
SMD: Standardized mean difference
tRNS: Transcranial random noise stimulation
