# Supplementary material for: A systematic review and meta-analysis of the effects of non-pharmacological interventions on quality of life in adults with multiple sclerosis
Source: Eur J Med Res. 2023 Aug 22;28:294. doi: 10.1186/s40001-023-01185-5 (PMC10463700; doi:10.1186/s40001-023-01185-5)
Supplement: Supplementary file 1 — Additional file 1. Detailed search strategy. [file 40001_2023_1185_MOESM1_ESM.docx]

# **Additional File 1**

*Detailed Search Strategy:*

Searching in SCOPUS - Results: 601

TITLE-ABS-KEY ( "Multiple Sclerosis" OR "encephalomyelitis disseminate" OR "demyelinating" ) AND TITLE-ABS-KEY ( " Health Status Questionnaire" OR "SF-36" OR " Multiple Sclerosis Quality of Life-54" OR " MSQOL-54" OR " Multiple sclerosis quality of life inventory" OR " MSQLI") AND ( LIMIT-TO ( DOCTYPE , "ar" ) ) AND ( LIMIT-TO ( LANGUAGE , "English" ) )

Searching in WEB OF SCIENCE - Results: 693

(TS=("multiple sclerosis" OR "encephalomyelitis disseminate" OR "demyelinating")) AND TS=(" Health Status Questionnaire" OR "SF-36" OR " Multiple Sclerosis Quality of Life-54" OR " MSQOL-54" OR " Multiple sclerosis quality of life inventory" OR "MSQLI") and Articles (Document Types) and English (Languages) and Articles (Document Types)

Searching in CINAHL-PLUS - Results: 37

"Multiple Sclerosis" AND ("Health Status Questionnaire" OR "SF-36" OR "Multiple Sclerosis Quality of Life-54" OR " MSQOL-54" OR "Multiple sclerosis quality of life inventory" OR "MSQLI")

Limiters - Publication Type: Journal Article; Age Groups: All Adult; Peer Reviewed; English Language; Exclude MEDLINE records; Human

Expanders - Add additional thesaurus terms to query; Apply equivalent subjects

Search modes - Boolean/Phrase

Searching in COCHRANE LIBRARY - Results: 354

("Multiple Sclerosis" OR "encephalomyelitis disseminate" OR "demyelinating") AND ( " Health Status Questionnaire" OR "SF-36" OR " Multiple Sclerosis Quality of Life-54" OR " MSQOL-54" OR " Multiple sclerosis quality of life inventory" OR " MSQLI") in Title Abstract Keyword - (Word variations have been searched)

Searching in OVID MEDLINE - Results: 589

(("Multiple Sclerosis/" or "encephalomyelitis disseminate" or "demyelinating") and (" Health Status Questionnaire" or "SF-36" or " Multiple Sclerosis Quality of Life-54" or " MSQOL-54" or " Multiple sclerosis quality of life inventory" or " MSQLI")).mp. [mp=title, abstract, original title, name of substance word, subject heading word, floating sub-heading word, keyword heading word, organism supplementary concept word, protocol supplementary concept word, rare disease supplementary concept word, unique identifier, synonyms] limit 1 to english language

Searching in EMBASE - Results: 556

(("Multiple Sclerosis" or "encephalomyelitis disseminate" or "demyelinating") and ("Health Status Questionnaire" or "SF-36" or " Multiple Sclerosis Quality of Life-54" or " MSQOL-54" or "Multiple sclerosis quality of life inventory" or " MSQLI")).mp. [mp=title, abstract, heading word, drug trade name, original title, device manufacturer, drug manufacturer, device trade name, keyword, floating subheading word, candidate term word] (human and english language and embase)
