# Supplementary material for: A systematic review and meta-analysis of the effects of non-pharmacological interventions on quality of life in adults with multiple sclerosis
Source: Eur J Med Res. 2023 Aug 22;28:294. doi: 10.1186/s40001-023-01185-5 (PMC10463700; doi:10.1186/s40001-023-01185-5)
Supplement: Supplementary file 2 — Additional file 2. Complete JBI critical appraisal checklist for all studies included in the meta-analysis. [file 40001_2023_1185_MOESM2_ESM.docx]

## **Additional File 2**

## *Complete JBI Critical appraisal checklist for all studies included in the meta-analysis:*

|  | 1. Was true randomization used for assignment of participants to treatment groups? | 2. Was allocation to treatment concealed? | 3. Were treatment groups similar at the baseline? | 4. Were participants blind to treatment assignment? | 5. Were those delivering treatment blind to treatment assignment? | 6. Were outcomes assessors blind to treatment assignment? | 7. Were treatment groups treated identically other than the intervention of interest? | 8. Was follow up complete and if not, were differences between groups in terms of their follow up adequately described and analyzed? | 9. Were participants analyzed in the groups to which they were randomized? | 10. Were outcomes measured in the same way for treatment groups? | 11. Were outcomes measured in a reliable way? (i.e disease specific QOL measure) | 12. Was appropriate statistical analysis used? | 13. Was the trial design appropriate, and any deviations from the standard RCT design (individual randomization, parallel groups) accounted for in the conduct and analysis of the trial? |
| --- | --- | --- | --- | --- | --- | --- | --- | --- | --- | --- | --- | --- | --- |
| Ahadi 2013(23) | Unclear | Unclear | Yes | No | No | No | Unclear | Yes | Yes | Yes | MS-QOL | Yes | Yes |
| Ahmadi 2010(24) | Unclear | Unclear | Yes | No | No | No | Unclear | Yes | Yes | Yes | MS-QOL | Yes | Yes |
| Ahmadi, Arastoo 2010(45) | Unclear | Unclear | Yes | No | No | No | Unclear | Yes | Yes | Yes | MS-QOL | Yes | Yes |
| Ashtari 2016(29) | Unclear | Unclear | No | Yes | Yes | Yes | Unclear | Yes | Yes | Yes | MS-QOL | Yes | Yes |
| Backus 2020(25) | Unclear | Unclear | Unclear | No | Yes | No | Yes | Yes | Yes | Yes | MS-QOL | Yes | Yes |
| Barclay 2019(26) | Unclear | Yes | Yes | No | No | Yes | Yes | Yes | Yes | Yes | MS-QOL | Yes | Yes |
| Bjarnadottir 2007(27) | Unclear | Unclear | Yes | No | No | Yes | Yes | No | No | Yes | SF-36 | Yes | Yes |
| Carter 2010(46) | Yes | Yes | Yes | No | No | No | Yes | Yes | Yes | Yes | MS-QOL | Yes | Yes |
| Dalgas 2010(33) | Yes | Yes | Yes | No | No | No | Yes | No | No | Yes | SF-36 | Yes | Yes |
| De Giglio 2015(37) | Yes | Yes | Yes | No | No | Yes | Unclear | No | Yes | Yes | MS-QOL | Yes | Yes |
| Etemadifar 2013(50) | Yes | Unclear | Yes | Yes | Yes | Yes | Yes | Yes | Yes | Yes | MS-QOL | Yes | Yes |
| Herbert 2018(35) | Yes | Yes | Yes | No | No | Yes | Yes | No | No | Yes | SF-36 | Yes | Yes |
| Jeong 2021(32) | Unclear | Unclear | Unclear | No | No | Yes | Yes | Yes | Yes | Yes | MS-QOL | Yes | Yes |
| Jongen 2019(48) | Yes | Yes | Yes | No | No | No | Yes | Yes | Yes | Yes | MS-QOL | Yes | Yes |
| Kargarfard 2012(47) | Yes | Yes | Yes | No | No | Yes | Yes | Yes | Yes | Yes | MS-QOL | Yes | Yes |
| Langeskov-Christensen 2021(34) | Yes | Yes | Yes | No | No | No | Yes | No | Yes | Yes | SF-36 | Yes | Yes |
| Momenabadi 2020(49) | Yes | Unclear | Yes | No | No | No | Yes | Yes | Yes | Yes | MS-QOL | Yes | Yes |
| Moravejolahkami 2020(41) | Yes | Yes | Yes | No | No | No | Unclear | No | Yes | Yes | MS-QOL | Yes | Yes |
| Namjooyan 2019(40) | Yes | Yes | Yes | Yes | Yes | Yes | Yes | No | Yes | Yes | MS-QOL | No | Yes |
| Nozari 2019(30) | Unclear | Unclear | Yes | Yes | Unclear | Yes | Yes | Unclear | Unclear | Yes | MS-QOL | Yes | Yes |
| O'Hara 2002(38) | Yes | Yes | Yes | No | No | Yes | Yes | No | No | Yes | SF-36 | Yes | Yes |
| Oken 2004(36) | Yes | Unclear | Yes | No | No | Yes | Yes | No | Yes | Yes | SF-36 | Yes | Yes |
| Pappalardo 2016(43) | Yes | Yes | Yes | No | No | Yes | Yes | Yes | Yes | Yes | SF-36 | Yes | Yes |
| Patti 2002(44) | Yes | Yes | Yes | No | No | Yes | Yes | Yes | Yes | Yes | SF-36 | Yes | Yes |
| Romberg 2005(28) | Unclear | Unclear | Yes | No | No | No | Yes | Yes | Yes | Yes | MS-QOL | Yes | Yes |
| Shinto 2008(31) | Unclear | Unclear | Yes | No | No | Yes | Yes | Yes | Yes | Yes | SF-36 | Yes | Yes |
| Siahpoosh 2018(51) | Yes | Yes | Yes | Yes | Unclear | Yes | Yes | Unclear | Yes | Yes | MS-QOL | Unclear | Yes |
| Stuijbergen 2003(39) | Yes | No | Yes | No | No | No | Yes | No | No | Yes | SF-36 | Yes | Yes |
| Vermöhlen 2017(42) | Yes | Yes | Yes | No | No | Yes | Yes | No | Yes | Yes | MS-QOL | Yes | Yes |
