# Supplementary material for: A systematic review and meta-analysis of the effects of non-pharmacological interventions on quality of life in adults with multiple sclerosis
Source: Eur J Med Res. 2023 Aug 22;28:294. doi: 10.1186/s40001-023-01185-5 (PMC10463700; doi:10.1186/s40001-023-01185-5)
Supplement: Supplementary file 3 — Additional file 3. Qualitative synthesis of studies not included in the meta-analysis. [file 40001_2023_1185_MOESM3_ESM.docx]

**Additional File 3**

*Qualitative synthesis of studies not included in the meta-analysis*

| Summary of the characteristics of qualitative studies not included in the meta-analysis investigating the effects of non-pharmacological interventions on health-related quality of life (HRQoL) - categorized by type of non-pharmacological intervention. | | | | | | | | | | |
| --- | --- | --- | --- | --- | --- | --- | --- | --- | --- | --- |
| Author/Year | Region | Sample size (Tx/Cx)^1^ | Participants | Mean age (range) | % Female | Group 1 | Group 2 | Duration | HRQoL tool | Summary of main outcome related to HRQoL |
| Physical Activity Interventions | | | | | | | | | | |
| Bansi(53) | Switzerland, Single Center | 52(ergometer 28/aquatic 24) | MS EDSS Score 1-6.5 | 51 | 67 | Cycle ergometer training for 30 minutes daily | Aquatic cycling for 30 minutes daily | 3 weeks | SF-36 | Both training groups showed increase in HRQoL in the mental and physical domains. |
| Gandolfi(54) | Italy, Single Center | 22(12,10) | MS  EDSS Score 1-6.5 | 50 | 72 | Patients performed RAGT for end effector systems training by placing feet on footplates and entering a harness where movements were controlled by robes attached to the harness. Each session was 40 minutes and occurred twice weekly | The SIBT practiced balance exercises of varying difficulty and were repeated under the sensory conditions: free vision, mask, and helmet. The tasks were aimed at postural control. Each session was 50 minutes twice weekly | 6 weeks | MSQOL-54 | There was no significant difference in HRQoL between the two training types. There was improvement in the SIBT physical health component overtime. |
| Khalil(55) | Jordan, Multi Center | 32(16,16) | MS relapsing remitting  EDSS Score 3-6.5 | 37 | 69 | VR training that included 10 levels of progressing difficulty, exercises including standing from a chair, squatting, reaching for objects, rising on toes, shifting their weight, and stepping over objects. These sessions were twice weekly plus one at home exercise per week. | Home exercise group were given a sheet of exercises to perform at home that encompassed exercises that were present in the VR training group. They were instructed to complete the exercises three times weekly | 6 weeks | SF-36 | The VR training group showed a statistically significant improvement in the MCS and PCS components of the SF-36 in comparison to the home exercise group. |
| Kerling(56) | Hannover Germany, Single Center | 60(30,30) | MS  EDSS Score 0-6 | 44 | 73 | Combined workout group (CWG) completed a combined endurance/resistance workout which consisted of 20 minutes cycling and then 20 minutes of resistance training twice weekly. | Endurance workout group (EWG) completed a 40-minute endurance program twice weekly. | 3 months | SF-36 | Both groups showed increased QoL in sub scales: role limitations due to physical limitations, general health perceptions, vitality, social functioning and mental health. Both groups did not have a statistically significant change in the physical components scores. |
| Munari(57) | Italy, outpatient Single Center | 17(RAGT-VR:8/RAGT:9) | MS  EDSS Score 3-6 | 54 | 59 | RAGT-VR: Robot assisted gait training with end effector system training with virtual reality walking trail simulation. Sessions were individualized 40-minute physiotherapy sessions twice weekly | RAGT: Robot assisted gait training with end effector system training without virtual reality. Sessions were individualized 40-minute physiotherapy sessions twice weekly. | 6 weeks | MSQOL-54 | Both groups showed improvements in the mental and physical component scores following intervention. |
| Pilutti(58) | Illinois, USA, outpatient Single Center | 12 | MS  EDSS Score 6-8 | 54 | 50 | Total-body recumbent stepper training (TBRST). Exercises for upper and lower body using arm levers and foot pedals. 30-minute sessions three times weekly. | Body weight–supported treadmill training (BWSTT)  Overhead pulley system supports exercise on a treadmill. 30-minute sessions three times weekly | 3 months | MSQOL-54 | Both interventions increased HRQoL. TBRST showed a smaller positive effect in comparison to BWSTT in both the mental and physical HRQoL measures |
| Solari(59) | Milan, Italy, Single Center | 50(27i,23c) | MS  EDSS Score 3-6.5 | 45 | 56 | Inpatient physical rehabilitation: 45-minute sessions twice daily with a trained physiotherapist for 3 weeks followed by home self-executed exercises for the remainder of the 15 weeks | Control: following a 1-day info session with a physiotherapist the patients performed home self-executed exercises for 15 weeks | 15 weeks | SF-36 | The PCS score of the study group were not statistically significant. The MCS score was statistically significant at 3 weeks and 9 weeks. |
| Behavioral & Psychological Interventions | | | | | | | | | | |
| Impellizzeri (60) | Reggio Calabria, Italy, outpatient Single Center | 30(15,15) | MS  EDSS Score 3-7 | 52 | 37 | Conventional cognitive behavioural therapy 6 times weekly in addition to neurologic music therapy (NMT) 3 times weekly | Control: conventional cognitive behavioural therapy 6 times weekly | 2 months | MSQOL-54 | The intervention group showed a statistically significant increase in the physical and mental composite scores post intervention |
| Plow(61) | Providence, RI, USA outpatient multi-center | 44 (IPR 22,GWI 20) | MS | 49 | 75 | Individualized physical rehabilitation (IPR) is four physical therapy sessions in addition to 3 phone calls to promote compliance with the activity. | Group wellness intervention (GWI) was 7 education sessions 2 hours each week for 7weeks with various educational modules focused on wellness. | 2 months | SF-36 | Both groups did not show a statistically significantly improvement in the SF-36 PCS or MSC scores at the 2 month follow-up time point. |
| Nutraceutical/Supplement Interventions | | | | | | | | | | |
| Weinstock-Guttman (62) | New York, USA, outpatient, multi-center | 27 (FO:13, OO:14) | Relapsing remitting MS  EDSS Score ⩽2.0 | 42 | 85 | Low fat diet supplemented with fish oil (FO) supplements | American Heart Association step I diet supplemented with olive oil (OO) | 12 months | SF-36 | Physical component score was higher in the FO group but benefits was seen in both groups. |
| Other Interventions | | | | | | | | | | |
| Salemi(62) | Palermo Italy, outpatient, single center | 17(9i,8c) | Relapsing remitting MS | 42 | 71 | Transcranial random noise stimulation (tRNS) of alternating current at 1.5 mA applied at random frequencies 100 to 640 Hz. tRNS was given daily for 2 weeks Monday to Friday for 15-minute intervals | Control group received sham tRNS which consisted of tRNS for 15s, and the remaining 15 minutes the tRNS was off. | 10 days | MSQOL-54 | The tRNS group showed benefit in the MSQoL-54 domains: change in health and role limitation due to physical problems |

1: In the Sample Size column ‘i’ refers to the intervention arm and ‘c’ is in reference to the control arm.
