# Supplementary material for: A systematic review and meta-analysis of the effects of non-pharmacological interventions on quality of life in adults with multiple sclerosis
Source: Eur J Med Res. 2023 Aug 22;28:294. doi: 10.1186/s40001-023-01185-5 (PMC10463700; doi:10.1186/s40001-023-01185-5)
Supplement: Supplementary file 4 — Additional file 4. Funnel plots of studies included in the meta-analysis. [file 40001_2023_1185_MOESM4_ESM.docx]

**Additional File 4**

*Funnel plots of studies included in the meta-analysis*


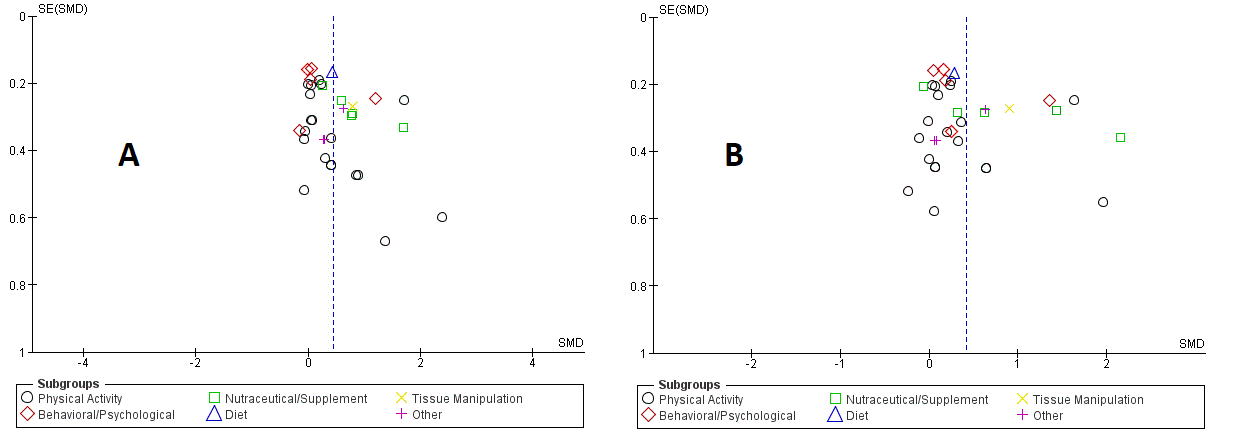

**A**: Funnel plots of studies included in the meta-analysis reporting effects on the physical health component of health-related quality of life domains. **B**: Funnel plots of studies included in the meta-analysis reporting effects on the mental health component of health-related quality of life domains.
